# Deciphering the molecular basis of mycobacteria and lipoglycan recognition by the C-type lectin Dectin-2

**DOI:** 10.1038/s41598-018-35393-5

**Published:** 2018-11-15

**Authors:** Alexiane Decout, Sandro Silva-Gomes, Daniel Drocourt, Emilyne Blattes, Michel Rivière, Jacques Prandi, Gérald Larrouy-Maumus, Anne-Marie Caminade, Beston Hamasur, Gunilla Källenius, Devinder Kaur, Karen M. Dobos, Megan Lucas, Iain C. Sutcliffe, Gurdyal S. Besra, Ben J. Appelmelk, Martine Gilleron, Mary Jackson, Alain Vercellone, Gérard Tiraby, Jérôme Nigou

**Affiliations:** 1 Institut de Pharmacologie et de Biologie Structurale, Université de Toulouse, CNRS, Université Paul Sabatier, 31077 Toulouse, France; 2 InvivoGen, Research Department, 31400 Toulouse, France; 3 Laboratoire de Chimie de Coordination, Université de Toulouse, CNRS, Université Paul Sabatier, 31077 Toulouse, France; 4 Department of Microbiology, Tumor and Cell Biology, Karolinska Institutet, 171 77, Stockholm, Sweden; 5 Biopromic AB, 171 65, Solna, Sweden; 6 Department of Medicine, Karolinska Institutet Solna 171 76, Stockholm, Sweden; 7 Mycobacteria Research Laboratories, Department of Microbiology, Immunology and Pathology, Colorado State University, Fort Collins, CO 80523-1682 USA; 8 Faculty of Health and Life Sciences, Northumbria University, Newcastle upon Tyne, NE1 8ST UK; 9 Institute of Microbiology and Infection, School of Biosciences, University of Birmingham, Edgbaston, Birmingham B15 2TT UK; 10 Department of Medical Microbiology and Infection Control, VU University Medical Center, 1081 BT Amsterdam, The Netherlands; 11 Present Address: Global Health Institute, Ecole Polytechnique Fédérale de Lausanne (EPFL), 1015 Lausanne, Switzerland; 12 Present Address: GlaxoSmithKline (GSK), Stevenage Herts, SG1 2NY UK; 13 Present Address: Innovative Medecine for Tuberculosis (iM4TB), Ecole Polytechnique Fédérale de Lausanne (EPFL), 1015 Lausanne, Switzerland; 14 Present Address: Medical Research Council Centre for Molecular Bacteriology and Infection, Imperial College London, London, SW7 2AZ UK; 15 Present Address: Massachusetts Supranational TB Reference Laboratory, University of Massachusetts Medical School, Jamaica Plain, MA 0213 USA

## Abstract

Dectin-2 is a C-type lectin involved in the recognition of several pathogens such as *Aspergillus fumigatus*, *Candida albicans*, *Schistosoma mansonii*, and *Mycobacterium tuberculosis* that triggers Th17 immune responses. Identifying pathogen ligands and understanding the molecular basis of their recognition is one of the current challenges. Purified *M*. *tuberculosis* mannose-capped lipoarabinomannan (ManLAM) was shown to induce signaling *via* Dectin-2, an activity that requires the (α1 → 2)-linked mannosides forming the caps. Here, using isogenic *M*. *tuberculosis* mutant strains, we demonstrate that ManLAM is a *bona fide* and actually the sole ligand mediating bacilli recognition by Dectin-2, although *M*. *tuberculosis* produces a variety of cell envelope mannoconjugates, such as phosphatidyl-*myo*-inositol hexamannosides, lipomannan or manno(lipo)proteins, that bear (α1 → 2)-linked mannosides. In addition, we found that Dectin-2 can recognize lipoglycans from other bacterial species, such as *Saccharotrix aerocolonigenes* or the human opportunistic pathogen *Tsukamurella paurometabola*, suggesting that lipoglycans are prototypical Dectin-2 ligands. Finally, from a structure/function relationship perspective, we show, using lipoglycan variants and synthetic mannodendrimers, that dimannoside caps and multivalent interaction are required for ligand binding to and signaling *via* Dectin-2. Better understanding of the molecular basis of ligand recognition by Dectin-2 will pave the way for the rational design of potent adjuvants targeting this receptor.

## Introduction

Innate immune recognition is based on the detection of microbial molecular structures by host pattern recognition receptors (PRRs)^1^. PRRs belong to several families, among which C-type lectins are specialized in the binding of sugar moieties, *via* carbohydrate recognition domains (CRD) that contain one or more calcium-dependent carbohydrate-binding sites^2^. Dectin-2 (Dendritic-Associated C-type lectin-2) is a C-type lectin that was initially identified on murine dendritic cells^3^, but was subsequently shown to be expressed on several cell types, including macrophages^4,5^. It is constituted of one CRD, a transmembrane domain and a short intracellular domain devoid of signaling motif^3^. Association of Dectin-2 with a FcRγ chain triggers the recruitment and phosphorylation of the tyrosine kinase Syk^4^, formation of the Card9/Malt1/Bcl10 complex^6^ and translocation of NF-κB to the nucleus, leading to the production of cytokines and chemokines, such as TNF-α^7^. Syk also activates the MAPK pathway through the phospholipase Cγ2 and drives the production of Th17 polarizing molecules^8,9^.

Dectin-2 is involved in the recognition of several pathogens, such as *Aspergillus fumigatus*, *Candida albicans* and *Schistosoma mansonii*, driving the production of pro-inflammatory cytokines and inducing protective immunity^7,10,11^. Indeed, the CRD of Dectin-2 exhibits specificity toward high mannose glycoconjugates containing Manα1-2Man motifs^12^, mainly found in fungi. The recognition of the yeast *Malassezia furfur* by Dectin-2 was shown to be mediated through an O-linked mannobiose-rich mannoprotein^13^. More recently, glycan array experiments indicated that the presence of Manα1-2Man motifs increased the binding to Dectin-2^14^ and, accordingly, the crystal structure of human Dectin-2 CRD complexed with Man_9_GlcNAc_2_ oligosaccharide revealed a canonical C-type primary monosaccharide binding site centered on a Ca^2+^ ion as well as a secondary binding site for a second mannose residue^15^.

**Figure 1:**
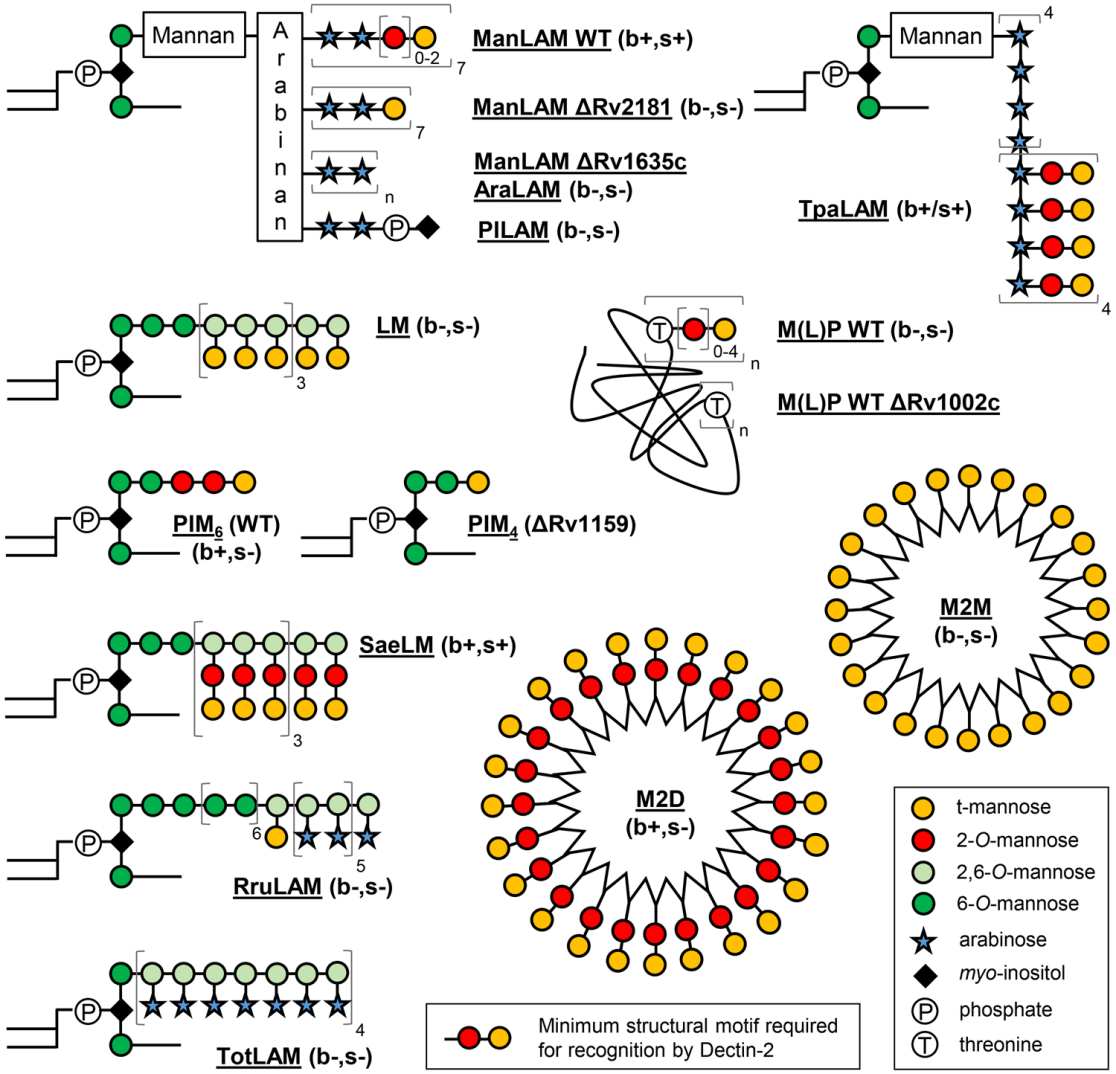
Chemical structure of the natural and synthetic mannoconjugates evaluated. AraLAM, lipoarabinomannan devoid of caps; LM: lipomannan; ManLAM, mannose-capped lipoarabinomannan; M(L)P, manno(lipo)proteins; M2M, M2D, second-generation mannodendrimers capped with mono- or di-mannosides respectively; PILAM, phospho-*my*o-inositol-capped lipoarabinomannan; PIM_4_, phosphatidyl-*myo*-inositol tetramannosides; PIM_6_: phosphatidyl-*myo*-inositol hexamannosides; RruLAM; LAM from *R*. *ruber*; SaeLM, LM from *S*. *aerocolonigenes*; TotLAM, LAM from *T*. *otitidis*; TpaLAM, LAM from *Ts*. *paurometabola*. Detailed structures are shown in Fig. S1. (b+/−, s+/−) indicates the ability (+) or not (−) of the mannoconjugates to bind (b) or induce signaling (s) *via* Dectin-2.

In addition to fungi and yeasts, Dectin-2 has been shown to play a key role in the detection of mycobacteria and induction of a protective immune response in mice^16^. Indeed, Dectin-2 knockout mice infected by the opportunistic pathogen *Mycobacterium avium* show a higher bacterial load than the wild-type mice. The lipoglycan mannose-capped lipoarabinomannan (ManLAM) produced by the slow-growing mycobacterial species, such as the human pathogen *Mycobacterium tuberculosis*, the vaccine strain *Mycobacterium bovis* BCG, or *M*. *avium*, but not by the non-pathogenic fast-growing species, such as *Mycobacterium smegmatis*^17,18^, was identified as a ligand of Dectin-2 when used as a purified molecule^16^. Recognition of purified ManLAM by Dectin-2 required the mannose caps^16^, which are mono-, (α1 → 2)-di- or (α1 → 2)-tri-mannosyl units present on the non-reducing ends of its arabinan domain^17^. However, the mycobacterial cell envelope contains a variety of complex glycoconjugates that bear similar structures, such as the phosphatidyl-*myo*-inositol hexamannosides (PIM_6_), lipomannan (LM) and manno(lipo)proteins (M(L)P) (Fig. 1), ubiquitously found in the envelope of mycobacteria^18–21^. Altogether, several questions remain unanswered. Is ManLAM a *bona fide* Dectin-2 ligand in the context of *M*. *tuberculosis* bacilli recognition? Are there other mycobacterial ligands of Dectin-2? Are bacterial lipoglycans prototypical ligands of this receptor? What is the precise molecular basis of ligand recognition?

Here, using on the one hand a set of *M*. *tuberculosis* isogenic mutant strains as well as purified and synthetic mannoconjugates, and on the other hand a combination of biochemical and cellular assays, we investigated: (i) the contribution of ManLAM and other mannoconjugates in *M*. *tuberculosis* recognition by Dectin-2, and (ii) the structure/function relationships and the molecular basis of ligand recognition by the receptor.

## Results

### ManLAM is the sole mycobacterial ligand triggering signaling *via* and mediating *M*. *tuberculosis* recognition by Dectin-2

**Figure 2:**
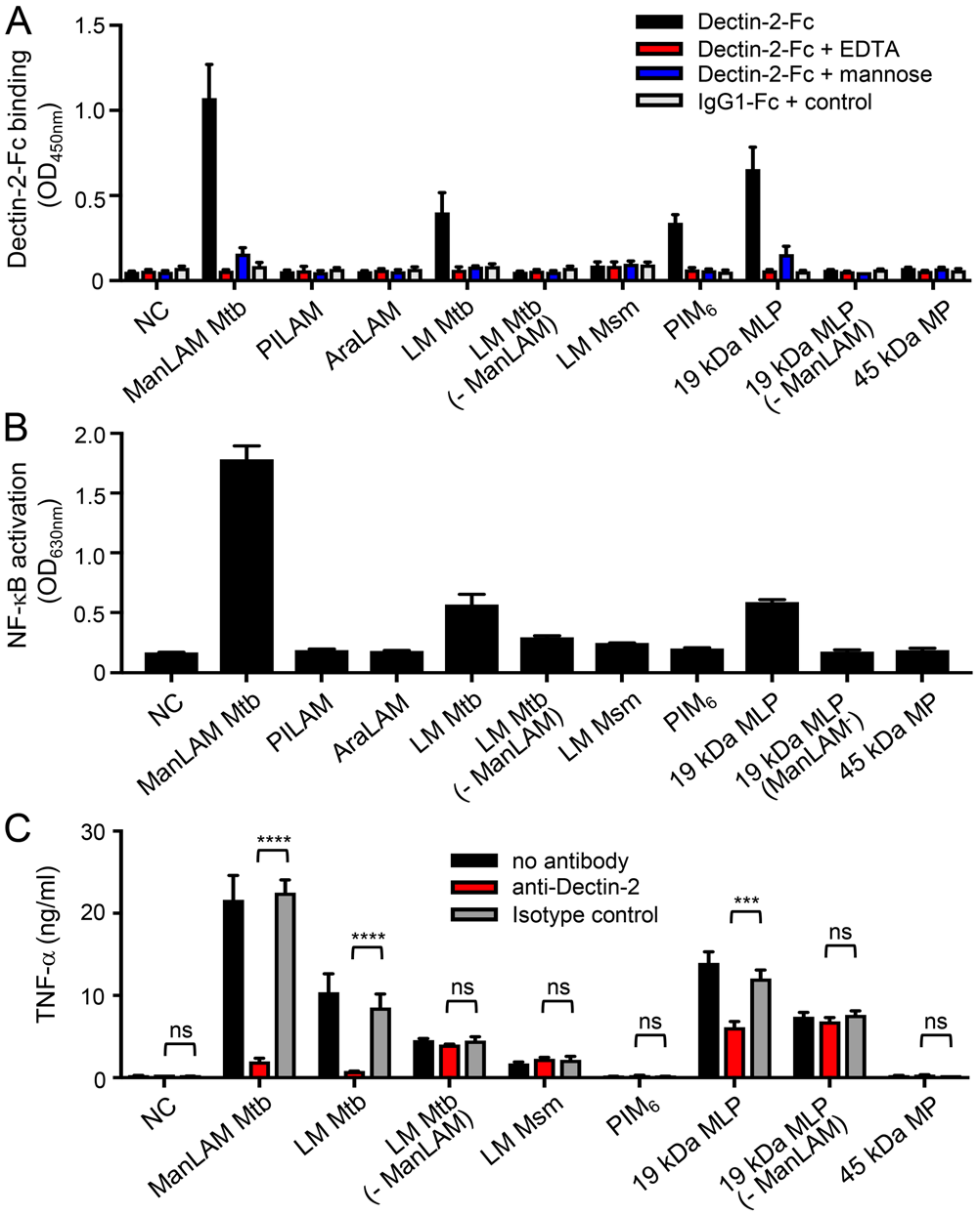
ManLAM, but not the other mycobacterial cell envelope mannoconjugates tested, induce cell signaling *via* Dectin-2. Mannoconjugates (1 µg in (**A**,**B**) 0.1 µg in (**C**)) were coated in 96-well plates and tested for their capacity to bind Dectin-2-Fc (**A**), and to induce NF-κB activation in HEK-Dectin-2 cells (**B**) and TNF-α production by BMDCs. (**A**) Dectin-2-Fc or IgG1-Fc control proteins (1 μg/ml) were pre-incubated or not with 20 mM EDTA or 40 mM mannose and allowed to react with the mannoconjugates for 2 h at RT. Bound proteins were detected using a biotin-conjugated anti-IgG Fcγ specific antibody and avidin-HRP, and reading O.D. at 450 nm. (**B**) HEK-Dectin-2 cells were stimulated for 24 h and NF-κB activation was determined by measuring alkaline phosphatase activity and reading O.D. at 630 nm. (**C**) BMDCs were stimulated for 24 h and TNF-α release in the supernatant was quantified by ELISA. Dectin-2 dependence was investigated by pre-incubating cells for 30 min at 37 °C with 5 μg/ml of anti-Dectin-2 or rIgG2a isotype control antibodies. Data show mean ± SEM. Mtb, *M*. *tuberculosis*; Msm, *M*. *smegmatis*; NC, non-coated; - ManLAM, ManLAM-depleted.

In order to first evaluate whether LM, PIM_6_ or M(L)P might constitute additional mycobacterial ligands of Dectin-2, we investigated the capacity of these compounds, purified from different strains, to (i) bind a soluble form of human Dectin-2 receptor (Dectin-2-Fc), (ii) induce NF-κB activation in HEK cells expressing murine Dectin-2 (HEK-Dectin-2) and a NF-κB-inducible reporter system (secreted alkaline phosphatase), and (iii) induce Dectin-2-dependent production of cytokines by murine bone marrow-derived dendritic cells (BMDCs). In agreement with published data^16^, ManLAM purified from *M*. *tuberculosis* efficiently bound Dectin-2-Fc (Fig. 2A), and induced NF-κB activation in HEK-Dectin-2 cells (Figs 2B and S2A) as well as a Dectin-2-dependent TNF-α production by BMDCs, as demonstrated by antibody blocking experiments (Fig. 2C). In contrast, but as expected, phosphoinositol-capped (PILAM) from *Mycobacterium fortuitum*^22^ and AraLAM from *Mycobacterium chelonae*^23^, which are devoid of mannose caps (Fig. 1)^17–19^, failed to do so (Fig. 2). *M*. *tuberculosis* LM bound and induced signaling *via* Dectin-2, although to a much weaker extent than ManLAM from the same strain (Figs 1 and S2A), while surprisingly *M*. *smegmatis* LM was completely inactive (Fig. 2). Only subtle differences in the mannan core ramification and acylation degrees between the structure of *M*. *tuberculosis* and *M*. *smegmatis* LM have been described to date^19^ and are unlikely to explain the difference in activity. However, the LAM and LM purification procedure involves a gel permeation chromatographic step to separate both lipoglycans^24,25^, which is not highly resolving, and yields preparation with a slight cross-contamination between the compound fractions. Whereas a contamination of *M*. *smegmatis* LM with PILAM from the same species would have no impact since PILAM is not a ligand of Dectin-2, a contamination of *M*. *tuberculosis* LM by ~1% of the strong agonist ManLAM would explain the activity observed for the former (Fig. S2A). Biochemical analysis of the *M*. *tuberculosis* LM batch used indeed confirmed a slight contamination by ManLAM, as demonstrated by the detection of arabinose after total acid hydrolysis (Fig. S2B). Accordingly, degradation of contaminating ManLAM by selective mild acid hydrolysis of its arabinan domain completely abrogated *M*. *tuberculosis* LM binding to and signaling *via* Dectin-2 (Fig. 2), while it did not affect LM ability to activate TLR2 (Fig. S2C). PIM_6_ bound weakly to Dectin-2-Fc (Fig. 2A), but failed to induce signaling in HEK-Dectin-2 cells or BMDCs (Fig. 2B and C). We finally tested two of the most abundant M(L)P purified from *M*. *tuberculosis*: the 19 kDa MLP (LpqH) and 45 kDa MP (Apa)^20,26,27^. The 19 kDa MLP, but not 45 kDa MP, bound and slightly induced signalling *via* Dectin-2 (Fig. 2). However again, depletion of ManLAM by anti-LAM antibody treatment completely abrogated the 19 kDa MLP Dectin-2-dependant activity (Fig. 2), while 19 kDa MLP remained potent at stimulating TLR2 (Fig. S2D). Altogether, although PIM_6_ weakly bound to Dectin-2, among all the mannoconjugates tested, ManLAM appears to be the sole mycobacterial ligand able to induce signaling *via* this receptor.

**Figure 3:**
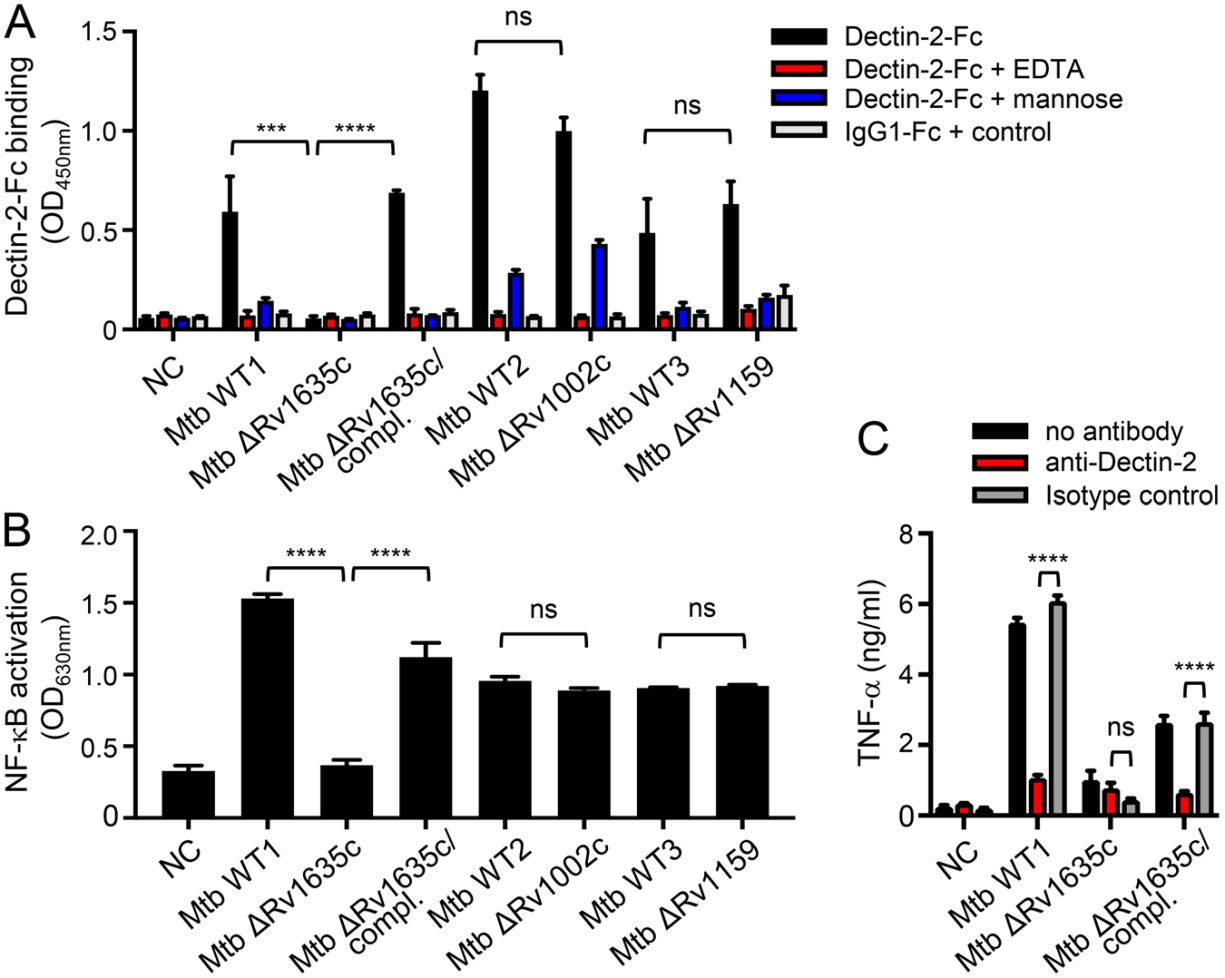
ManLAM is the sole ligand mediating *M*. *tuberculosis* recognition by Dectin-2. The indicated *M*. *tuberculosis* (Mtb) strains (10^6^ bacilli in (**A**) 1 µg lysate in (**B** and **C**)) were tested for their capacity to bind Dectin-2-Fc (**A**), and to induce NF-κB activation in HEK-Dectin-2 cells (**B**) and TNF-α production by BMDCs (**C**). Conditions are the same as in Fig. 2. Data show mean ± SEM. compl., complemented; NC, non-coated.

In order to evaluate the contribution of ManLAM, and other possible ligands, to *M*. *tuberculosis* recognition by Dectin-2, we investigated the capacity of different mutant strains to bind or induce signaling *via* the receptor. A *M*. *tuberculosis* ΔRv1635c/CapA mutant strain, which produces a LAM devoid of mannose caps (Fig. 1) while other mannoconjugates remain intact^28,29^, completely failed, in contrast to the wild-type and complemented strains, to bind Dectin-2-Fc (Fig. 3A). However, a *M*. *tuberculosis* ΔRv1159/PimE mutant strain, which is impaired for the production of PIM_6_ and accumulates PIM glycoforms devoid of (α1 → 2)-linked units^30^ (Figs 1 and S3), and a *M*. *tuberculosis* ΔRv1002c/PMT mutant strain, which is deficient for protein O-mannosylation^20^ (Fig. 1), bound Dectin-2-Fc as efficiently as their wild-type counterparts (Fig. 3A). Finally, the capacity of a bacterial lysate to induce NF-κB activation in HEK-Dectin-2 cells, as well as a Dectin-2-dependent TNF-α production by BMDCs, was fully abrogated in the ΔRv1635c/CapA mutant strain (Fig. 3B and C).

Thus, ManLAM is concluded to be the sole ligand mediating *M*. *tuberculosis* recognition by Dectin-2.

### Dimannoside caps are required for ManLAM, and related lipoglycans, binding to and signaling *via* Dectin-2

**Figure 4:**
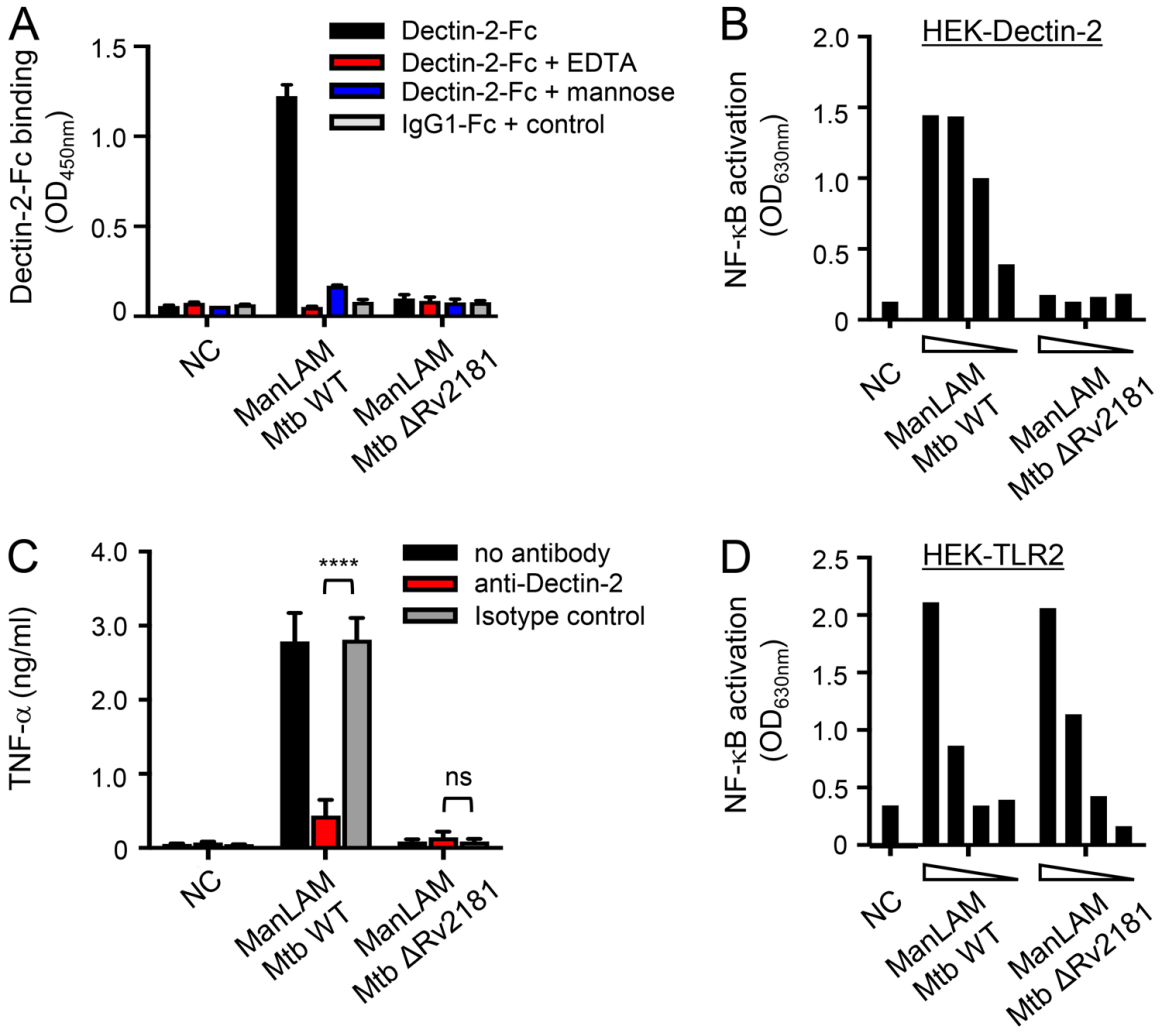
Dimannoside caps are required for ManLAM binding to and signaling *via* Dectin-2. ManLAM (1 µg in (**A**) from 300 to 10 ng in (**B** and **D**) 0.1 µg in (**C**)) purified from *M*. *tuberculosis* wild-type or Δ*Rv2181* mutant strains were tested for their capacity to bind Dectin-2-Fc (**A**), to induce NF-κB activation in HEK-Dectin-2 cells (**B**) and HEK-TLR2 cells (**D**), and to induce TNF-α production by BMDCs (**C**). Conditions are the same as in Fig. 2. Data show mean ± SEM. NC, non-coated.

The mannose caps are necessary for ManLAM recognition by Dectin-2^16^. However, these motifs are heterogeneous and consist of mono-, (α1 → 2)-di- or (α1 → 2)-tri-mannosyl units^17^. In order to assess the impact of the mannose cap length, we used ManLAM purified from a *M*. *tuberculosis* ΔRv2181 mutant strain, which harbours single mannose residues at the non-reducing arabinan termini instead of (α1 → 2)-linked oligomannosides^31^ (Fig. 1). In contrast to wild-type ManLAM, ManLAM capped with single mannose units failed to bind or induce signaling *via* Dectin-2, while, as expected^32,33^, it was still able to activate TLR2 (Fig. 4). Thus, at least dimannoside caps are required for ManLAM recognition by Dectin-2, in agreement with the reported i) glycan array analyses showing selective binding of the Dectin-2 CRD to glycans containing (α1 → 2)-linked dimannoside epitopes^14,15^, and ii) the crystal structure of the CRD in complex with a mammalian-type high-mannose Man_9_GlcNAc_2_ revealing two monosaccharide binding sites that allow the interaction of dimannosides^15^.

**Figure 5:**
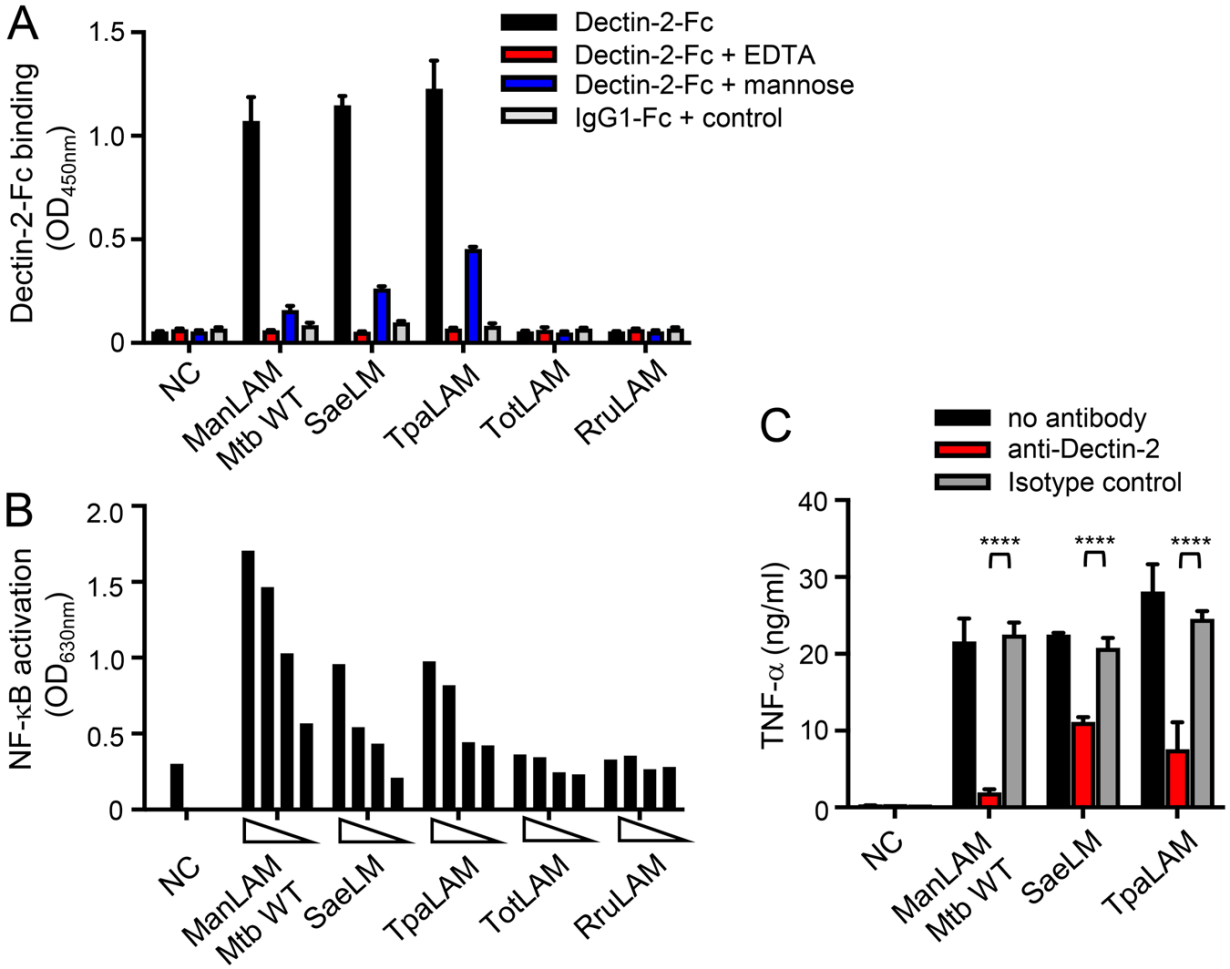
Actinobacteria lipoglycans bearing (α1 → 2)-linked dimannoside caps bind and induce signaling *via* Dectin-2. Lipoglycans (1 µg in (**A**) from 300 to 10 ng in (**B**) 0.1 µg in (**C**)) were tested for their capacity to bind Dectin-2-Fc (**A**), and to induce NF-κB activation in HEK-Dectin-2 cells (**B**) and TNF-α production by BMDCs (**C**). Conditions are the same as in Fig. 2. Data show mean ± SEM. NC, non-coated.

Interestingly, bacteria belonging to genera phylogenetically close to mycobacteria produce lipoglycans with structures related to LAM^32^. LAM from *Tsukamurella paurometabola* (TpaLAM)^34^ and LM from *Saccharotrix aerocolonigenes* (SaeLM)^35^ contain (α1 → 2)-linked dimannoside side chains, whereas LAM from *Turicella otitidis* (TotLAM)^36^ or *Rhodococcus ruber* (RruLAM)^37^ show single terminal mannose units only as in mycobacterial LM (Fig. 1). SaeLM and TpaLAM, but not TotLAM or RruLAM, were able to bind and induce signaling *via* Dectin-2 (Fig. 5), further supporting the structure/function relationship conclusions drawn with mycobacterial lipoglycans. However, if (α1 → 2)-linked dimannosides are required for the recognition by the CRD, they are not sufficient since both PIM_6_ and M(L)P fail to induce signaling *via* Dectin-2.

### Signaling *via* Dectin-2 relies on multivalent interaction

**Figure 6:**
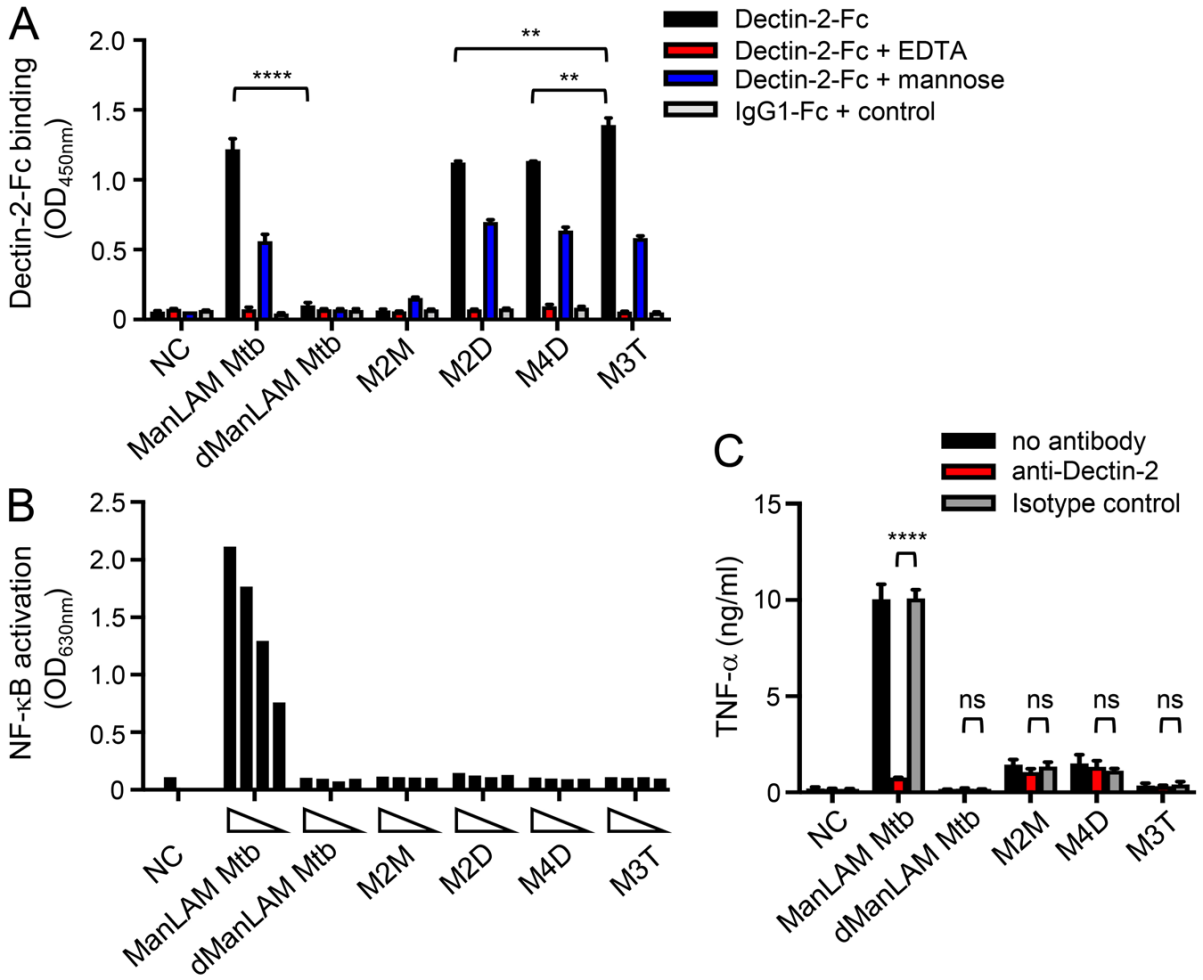
Mannodendrimers bind but do not induce signaling *via* Dectin-2. Mannoconjugates (1 µg in (**A**) from 300 to 10 ng in (**B**) 0.1 µg in (**C**)) were tested for their capacity to bind Dectin-2-Fc (**A**), and to induce NF-κB activation in HEK-Dectin-2 cells (**B**) and TNF-α production by BMDCs (**C**). Conditions are the same as in Fig. 2. Data show mean ± SEM. dManLAM, deacylated ManLAM; M2M, M2D, second-generation mannodendrimers capped with mono- or di-mannosides respectively; M3T, third-generation mannodendrimer capped with trimannosides; M4D, fourth-generation mannodendrimer capped with dimannosides; NC, non-coated.

High avidity recognition by C-type lectins relies on multivalent binding that accumulates the strength of the multiple low affinities of the interaction between individual CRDs and oligosaccharides^38,39^. We previously found that high avidity recognition of purified ManLAM by the C-type lectins Mannose Receptor or DC-SIGN requires a ManLAM supramolecular organization induced by the aggregation of fatty acids in aqueous solution^21,40,41^. Indeed, although fatty acids do not directly interact with the receptor, they are involved in the 3D conformational presentation of the mannose caps and are required for high avidity binding of ManLAM. Accordingly, deacylation of ManLAM completely abrogated its ability to bind and induce signaling *via* Dectin-2 (Fig. 6). With the aim to mimic the bioactive supramolecular structure of ManLAM, we recently designed and chemically synthesized a set of mannodendrimers, made of poly(phosphorhydrazone) dendrimers grafted with (α1 → 2)-linked mannose caps (Figs 1 and S1), that bind and induce signaling *via* DC-SIGN as efficiently as the natural *M*. *tuberculosis* molecule^42^. Whatever the dendrimer generation, mannodendrimers grafted with dimmanoside or trimannoside caps bound Dectin-2-Fc as efficiently as ManLAM, whereas mannodendrimer with monomannoside caps did not (Fig. 6A), confirming again with the use of synthetic compounds that (α1 → 2)-linked dimannosides are required for the recognition by the CRD. However, none of the mannodendrimers was able to activate Dectin-2 signaling in reporter cells (Fig. 6B) or BMDCs (Fig. 6C), indicating that the ligand 3D conformational requirements for high avidity recognition by DC-SIGN and Dectin-2 differ.

## Discussion

C-type lectins play a key role in the immune system with functions including cell adhesion, glycoprotein turnover or pathogen recognition based either on recognition of endogenous mammalian glycans or on binding to glycans on micro-organisms^2^. The spatial arrangement of CRDs in C-type lectin oligomers, although still poorly understood, has long been recognized as important in determining specificity for pathogen glycans, especially as the selectivity of the CRDs was initially thought to be low, by binding only the terminal monosaccharides of glycans^2,43^. However, recent studies have revealed that several C-type lectin CRDs comprise extended binding sites that can interact with disaccharide or trisaccharide units in terminal, but also possibly in internal, positions^15,39,44^. The geometry, organization and amino-acid composition of the extended binding site lead to a selectivity towards, or preclude an efficient binding of, specific classes of oligosaccharides (containing given monosaccharides and linkages: position, anomeric configuration)^15^. However, if the mechanisms of glycans binding to CRDs are increasingly well understood, how it leads to initiation of C-type lectin-associated signaling pathways remains almost unknown^2,45^.

The recently published crystal structure of human Dectin-2 CRD complexed with Man_9_GlcNAc_2_ oligosaccharide has revealed an extended binding site providing the molecular basis for binding of Manα1-2Man in external or internal positions of glycans^15^, structures that are found in several pathogens, such as fungal mannans, certain bacterial lipopolysaccharides, or mycobacterial mannose-capped LAM. Accordingly, we show here that effective binding of Dectin-2 to ManLAM or synthetic mannodendrimers requires (α1 → 2)-linked dimannoside caps. Interestingly, binding was increased by addition of a third (α1 → 2)-linked mannosyl unit, as observed for mannodendrimers (M3T *vs* M4D and M2D), in agreement with the crystal structure that shows space to model an additional α-linked mannose residue attached to the 2-OH group of the secondary site mannose residue (at the non-reducing end)^15^. However, substitution of the 6-OH group of the mannosyl unit bearing the (α1 → 2)-linked mannosyl unit at the non-reducing end impairs the binding, most probably because of a steric hindrance, as demonstrated by the inactivity of mycobacterial LM in contrast to SaeLM. We wish here to highlight the observation that traces amount of the highly potent Dectin-2 ligand ManLAM were sufficient to confer activity to other compounds purified from *M*. *tuberculosis* cell envelope (LM, 19 kDa MLP). When working with ManLAM-producing bacteria, one has thus to be cautious regarding compound purity. Surprisingly, M(L)P did not bind Dectin-2 although they bear (α1 → 2)-linked oligomannosides. In addition, PIM_6_, which weakly binds, but also mannodendrimers, which strongly bind Dectin-2-Fc, at least as efficiently as ManLAM, failed to induce signaling. This probably results from their inability to establish adequate multivalent interactions with Dectin-2, which are required for efficient high avidity binding to and triggering of intracellular signaling *via* the membrane-expressed receptor. Mannodendrimers were previously designed and chemically synthesized to mimic the bioactive supramolecular structure of ManLAM, and to bind and induce signaling *via* DC-SIGN^42^. Third-generation poly(phosphorhydrazone) mannodendrimers were sufficient to induce signaling *via* DC-SIGN as efficiently as ManLAM^42^. However, in the present study, even a fourth-generation poly(phosphorhydrazone) mannodendrimer was not able to trigger signaling *via* Dectin-2. Increasing the generation is not likely to make any improvement. Other dendrimer scaffolds, with a different geometry, should rather be tested to try to obtain an adequate mutivalency with Dectin-2. How ligand binding initiates intracellular signaling remains arguably the most poorly understood mechanistic aspect of C-type lectin function^2^. Ligand avidity is certainly a key parameter, but apparently not sufficient. Mannodendrimer binding to DC-SIGN was evaluated in our previous study using a set of bioassays, including inhibition of HEK293 cells expressing wild-type DC-SIGN protein binding to mannan-coated microplates^42^. Interestingly, we observed that the mannodendrimer IC_50_ values were not completely sufficient for prediction of their capacity to induce DC-SIGN signaling in human monocyte-derived dendritic cells (i.e. inhibition of pro-inflammatory cytokine production by LPS-stimulated cells). Here again, binding to Dectin-2 was not predictive of the capacity to induce signaling. Clustering of receptors by ligand binding is likely to be important for signaling initiation, but so far little is known about the oligomeric state of Dectin-2 or stoichiometry of the complexes that form with FcRγ^2^.

To determine the Dectin-2 ligand(s) involved in *M*. *tuberculosis* recognition, we used a set of knockout mutant strains. Indeed, ligands identified using purified molecules may not be accessible or relevant in the bacterial envelope^46–48^. In contrast, physiological ligands of C-type lectins might poorly bind the receptor in a solid phase binding assay because of a different clustering. Finally, unsuspected Dectin-2 ligand(s) might be involved in *M*. *tuberculosis* recognition. However, a *M*. *tuberculosis* ΔRv1635c/CapA mutant strain, which produces a LAM devoid of mannose caps, completely failed, in contrast to the other mutant strains tested, to bind Dectin-2, indicating that ManLAM is the sole ligand mediating *M*. *tuberculosis* recognition by Dectin-2. PIM_6_, which binds Dectin-2-Fc but does not induce signaling *via* the receptor, is not involved in whole bacilli recognition by Dectin-2 in reporter cells or BMDCs. Although we cannot completely exclude that PIM_6_-Dectin-2 interaction may play a role in other cellular contexts, this means that Dectin-2 can sense slow-growing mycobacteria (which are mostly the pathogenic ones) but not fast-growing mycobacteria, which produce LAM devoid of mannose caps. This property is unique so far among C-type lectin receptors, which show different specificities towards mycobacteria. Indeed, Mannose Receptor can recognize several purified mycobacterial mannoconjugates, such as ManLAM, LM, PIM and the 19 kDa MLP and 45 kDa MP, some of them (LM, PIM and glycoproteins in general) being also produced by fast-growing mycobacteria. Accordingly, Mannose Receptor binds both slow- and fast-growing mycobacteria. DC-SIGN recognizes the same purified mycobacterial mannoconjugates. However, surprisingly, it binds species of the *M*. *tuberculosis* complex only^46^. The molecular basis of this selective binding is not yet fully understood^46–48^.

Beyond mycobacteria, our data also suggest that Dectin-2 might be involved in the detection of the human opportunistic pathogen *Ts*. *paurometabola*, with some strains of the species reported to cause lung infection, lethal meningitis, and necrotizing tenosynovitis^49^.

Knowledge of the mechanism of carbohydrate recognition by C-type CRDs is now becoming sufficient that glycomimetic drugs can be envisaged^2^. The recent discovery that some C-type lectins can induce intracellular signaling and elicit cell-mediated immune responses has attracted interest in these receptors and their ligands in the field of adjuvants and immunomodulation^45,50^. Indeed, there is a growing interest in the development of vaccine adjuvants that direct robust Th1 and Th17 responses to subunit vaccines. An analogue of the mycobacterial trehalose-6,6′-dimycolate ligand of Mincle, trehalose-6,6′-dibehenate (TDB), formulated with dimethyldioctadecylammonium was reported to promote long-lived *M*. *tuberculosis*-specific T-cell responses in humans^51^ and completed phase I clinical trial^52^. Addition of a newly identified Dectin-2 ligand, the glycoprotein *Blastomyces* Eng2, to a pan-fungal subunit vaccine was recently shown to prime large numbers of Ag-specific Th17 and Th1 cells, augment activation and killing of fungi, and protect mice from lethal fungal challenge^53^. Moreover, Dectin-2 activation by ManLAM was found to trigger limited inflammatory responses that could be beneficial for the adjuvantation of therapeutic vaccines for infectious diseases or cancer^16^. Therefore, better understanding the molecular basis of ligand recognition by Dectin-2 will pave the way for the rational design of potent adjuvants targeting this receptor^54^.

## Materials and Methods

### Mannoconjugates and mycobacterial strains

Lipoglycans and lipoproteins were purified as previously described; ManLAM Mtb, LM Mtb and PIM_6_ from *M*. *tuberculosis* H37Rv, AraLAM from *M*. *chelonae*, PILAM from *M*. *fortuitum*, LM Msm from *M*. *smegmatis*^46,55–57^; TpaLAM from *Ts*. *paurometabola*^34^; SaeLM from *S*. *aerocolonigenes*^35^; TotLAM from *T*. *otitidis*^36^; RruLAM from *R*. *ruber*^37^; the native 19 kDa lipoprotein^58^ and 45 kDa mannoprotein^59^ from *M*. *tuberculosis* H37Rv; ManLAM Mtb ΔRv2181 from *M*. *tuberculosis* H37Rv*ΔRv2181*^31^. Bacterial strains were grown under the following biosafety conditions: *M*. *tuberculosis* strains (level 3), *M*. *chelonae*, *M*. *fortuitum* and *T*. *otitidis* (level 2), other bacterial species (level 1). Mannodendrimers were chemically synthesized as previously reported^42^.

Traces of ManLAM were removed from the purified 19 kDa mannolipoprotein solution by immunoprecipitation, using magnetic beads coated with an anti-LAM antibody^60^. Beads (500 µg; ~10 µg antibody) were washed twice with PBS, added to 100 µl of the mannolipoprotein solution (at 1 mg/mL) and incubated for 1 h at room temperature. Using a magnet, the LAM-free supernatant was removed and used for subsequent experiments. ManLAM present in *M*. *tuberculosis* LM preparation was degraded by mild acid hydrolysis (0.1 M HCl for 20 min at 110 °C) that selectively depolymerizes the arabinan domain while keeping intact the lipomannan core^34^.

*M*. *tuberculosis* H37Rv*ΔRv1635c*^28^, *M*. *tuberculosis* H37Rv*ΔRv1002c*^20^, *M*. *tuberculosis* H37Rv*ΔRv1159* (see below), and corresponding wild-type strains were grown as surface pellicle in 7H9 medium supplemented with ADC. For binding experiments to Dectin-2-Fc (see below), bacteria were dissociated by gentle shaking for 30 s with 4-mm glass beads and numbered with a Thoma cell counting chamber. To prepare a bacterial lysate containing lipoglycans (LAM, LM and PIM_6_) and lipoproteins, mycobacteria were delipidated by several extractions with CHCl_3_/CH_3_OH (1:1, v/v). Delipidated cells were then disrupted by sonication and further extracted by refluxing in 50% ethanol at 65 °C^46^. Ethanol/water extract was dried and used in subsequent experiments.

### Construction of a *M*. *tuberculosis* H37RvΔ*Rv1159* mutant

The Ts/sacB method was used to achieve allelic replacement at the *pimE* (*Rv1159*) locus of *M*. *tuberculosis* H37Rv (ATCC 25618)^61^. *pimE* and flanking regions were PCR-amplified using the pair of primers Rv1159.5 (5′-ggcggcgggtgcgggttccgc-3′)/Rv1159.6 (5′-ccaagttgacggcggccaccg-3′) and a disrupted allele was obtained by replacing 964 bp of the coding sequence of this gene bracketed between two SmaI sites by the Kan cassette from pUC4K (Amersham Pharmacia Biotech). Mutant clones were confirmed by PCR using the set of primers Rv1159.1 (5′-CCCGGCCCATATGTGCCGCACCCTGATCGAC-3′) and Rv1159.2 (5′-CCCAAGCTTATTGGCCATGCGCCGCGGCC-3′). Allelic replacement at the *pimE* locus of eight candidate mutant clones was confirmed by PCR (Fig. S3A). Negative ion mode MALDI-TOF-MS analyses of the PIM content of one of the mutants, performed as previously described^62^, revealed a complete absence of PIM_6_ acyl-forms with a concomitant increase in PIM_4_ acyl-forms (Fig. S3B), indicating that the disruption of *pimE* had the same effects on polar PIM synthesis in *M*. *tuberculosis* as the inactivation of the orthologous gene (MSMEG_5149) in *M*. *smegmatis*^30^.

### Monosaccharide analysis

Mannoconjugates were submitted to strong acid hydrolysis with 2 M trifluoroacetic acid at 110 °C for 2 h and then dried under speed-vacuum. The resulting monosaccharides were derivatized for 90 min at 55 °C using a solution of 0.2 M 1-aminopyrene-3,6,8-trisulfonate (APTS) in 15% acetic acid and 1 M sodium cyanoborohydride solution dissolved in tetrahydrofuran. The APTS-labelled monosaccharides were suspended in water and subjected to analysis by capillary electrophoresis monitored by laser-induced fluorescence, as previously described^63^.

### Binding of Dectin-2-Fc

Mannoconjugates (1 μg/well in isopropanol) or mycobacteria (heat-inactivated, 10^6^/well in isopropanol) were coated on 96-wells Maxisorp plates (Nunc). Dectin-2-Fc, a soluble form of the human Dectin-2 receptor, was constructed by fusing the C-terminal extracellular domain of human Dectin-2 (aa 42–209) to the C-terminus of an engineered human IgG1 Fc domain. A soluble form of the murine Dectin-1 receptor fused to the same human IgG1-Fc domain was used as a non-relevant protein control (IgG1 Fc control). hDectin-2-Fc and IgG1-Fc control proteins were expressed in CHO cells and purified by G protein affinity chromatography. Human IgG1-Fc control or Dectin-2-Fc fusion proteins (1 μg/ml in PBS, 1 mM CaCl_2_, 1% BSA) were pre-incubated or not with 20 mM EDTA or 40 mM mannose (Sigma) and were allowed to react with mannoconjugates or bacterial cells for 2 h at RT (in 50 μl). Wells were washed once with PBS and the bound Fc fusion proteins were detected using biotin-conjugated anti-human IgG Fcγ specific antibodies (eBioscience) and avidin-horseradish peroxidase (eBioscience).

### Dectin-2 and TLR2 reporter cell lines experiments

The HEK-Blue^TM^ mDectin-2 and HEK-Blue^TM^ hTLR2 (InvivoGen), derivatives of HEK293 cells that stably express the murine Dectin-2 or human TLR2 genes respectively, along with a NF-κB-inducible reporter system (secreted alkaline phosphatase) were maintained in Dulbecco’s modified Eagle’s medium (DMEM, Gibco) containing 10% Fetal Bovine Serum (FBS, Gibco) 4.5 g/l glucose, 2 mM L-glutamine, 100 U/ml penicillin, 100 μg/ml streptomycin (Sigma) and 100 μg/ml zeocin, 200 μg/ml hygromycin, 10 μg/ml blasticidin, 1 μg/ml puromycin and 50 μg/ml mofetil (all from InvivoGen). Mannoconjugates (1 μg/well in isopropanol, except in Figs 4B,D, 5B, 6B and S2C, from 300 ng to 10 ng/well in isopropanol, in Fig. S2D from 20 to 0.2 ng/well) or bacterial cells lysate (1 µg/well in isopropanol) were added to 96-well plates, followed by evaporation of the solvents as previously described. Reporter cells (5 × 10^4^/well) were stimulated for 24 h, after which alkaline phosphatase activity was measured by mixing 20 μl of the culture supernatant and 180 μl of Quanti-Blue^TM^ (InvivoGen), and reading O.D. at 630 nm.

### Generation and activation of murine bone marrow-derived dendritic cells

All methods were carried out in accordance with the Centre National de la Recherche Scientifique guidelines and regulations for housing and care of laboratory animals. All experimental protocols were approved by the Structure chargée du bien-être animal (no. 2015.Ni.15). Bone marrow cells were flushed from the tibias and femurs of C57BL/6 mice (Janvier) with 5 ml of cold DMEM. The cell suspension was cultured at a density of 10^6^ cells/ml in Iscove’s modified Dulbecco’s medium (IMDM, Lonza) supplemented with 10% FBS, 100 U/ml penicillin, 100 μg/ml streptomycin, 50 μM 2-mercaptoethanol and 10% J558 cell conditioned medium (as a source of GM-CSF). On day 3, fresh medium containing GM-CSF was added and on day 6 one half of the medium was renewed. BMDCs were harvested and used on day 8. They were distributed in 96-well plates at 2 × 10^5^ cells/well to wells previously coated with mannoconjugates (0.1 µg/well) or bacterial cells lysate (1 µg/well) as described above. After 18 h, TNF-α was assayed in the culture supernatant using a commercially available kit (eBioscience). To investigate Dectin-2 dependence, BMDCs were pre-incubated for 30 min at 37 °C with 5 μg/ml of anti-mDectin-2 antibody (clone 11E4, Life Technology) or isotype control (rIgG2a, eBiosciences).

### Statistical analysis

Data are expressed as mean ± SEM and were analyzed using Two-way analysis of variance followed by Tukey test to determine significant differences between samples.

## Electronic supplementary material

Supplementary Figures
