# Supplementary material for: Deciphering the molecular basis of mycobacteria and lipoglycan recognition by the C-type lectin Dectin-2
Source: Sci Rep. 2018 Nov 15;8:16840. doi: 10.1038/s41598-018-35393-5 (PMC6237770; doi:10.1038/s41598-018-35393-5)
Supplement: Supplementary file 1 — Supplementary Figures [file 41598_2018_35393_MOESM1_ESM.pdf]

**SUPPLEMENTARY FIGURES for**

# Deciphering the molecular basis of mycobacteria and lipoglycan recognition by the C-type lectin Dectin-2

**Alexiane Decout, Sandro Silva-Gomes, Daniel Drocourt, Emilyne Blattes, Michel Rivière, Jacques Prandi, Gérald Larrouy-Maumus, Anne-Marie Caminade, Beston Hamasur, Gunilla Källenius, Devinder Kaur, Karen M. Dobos, Megan Lucas, Iain C. Sutcliffe, Gurdyal S. Besra, Ben J. Appelmelk, Martine Gilleron, Mary Jackson, Alain Vercellone, Gérard Tiraby & Jérôme Nigou**

**Figure S1. Structures of the lipoglycans (A) and mannodendrimers (B) used in this study.**

A) The correspondence between the structures and the symbols is shown for SaeLM.

B) Only one of the six branches substituting the N3P3 core is represented.

**Figure S2. Dectin-2 agonist activity in LM Mtb and 19 kDa MLP fractions relies on ManLAM.**

A, C, D) NF-κB activation induced by LM Mtb and 19 kDa MLP fractions in HEK-Dectin-2 or HEK-TLR2 cells. Mannoconjugates (from 1 to 1000 ng in A; from 300 to 10 ng in C; from 20 to 0.2 ng in D) were tested for their capacity to induce NF-κB activation in HEK-Dectin-2 (A) or HEK-TLR2 (C, D) cells. Conditions are the same as in Figure 2. - ManLAM, ManLAM-depleted.

B) Monosaccharide analysis of LM Mtb fraction. Partial capillary electropherograms of APTS derivatives of monosaccharides obtained from total acid hydrolysis of ManLAM Mtb (trace a) and LM Mtb (trace b). Peaks: Ara*, arabinose-APTS; Man*, mannose-APTS; C7*, internal standard, mannoheptose-APTS.

**Figure S3. Generation of a *pimE* knock-out mutant of *M. tuberculosis* H37Rv (A) and analysis of phosphatidyl-*myo*-inositol mannosides composition (B).**

A) Allelic replacement was confirmed by PCR using the set of primers (Rv1159.1 and Rv1159.2; see Materials and Methods for details). The wild-type 1,295 bp amplification signal (wt) is replaced by a 1,526-bp amplification signal in the mutant clones (Δ) due to the insertion of a 1.2 kb- kanamycin resistance cassette between the two SmaI restriction sites of *pimE*.

B) Negative ion mode MALDI-MS spectra of the total lipids from WT Mtb H37Rv and its *pimE* mutant (H37Rv Δ*pimE*). $PIM_2$, $PIM_4$, $PIM_6$, phosphatidyl-*myo*-inositol di-, tetramannosides; $PIM_6$: phosphatidyl-*myo*-inositol hexamannosides; $Ac_1PIM$, acylated PIM (3 acyl chains in total); $Ac_2PIM$, di-acylated PIM (4 acyl chains in total).

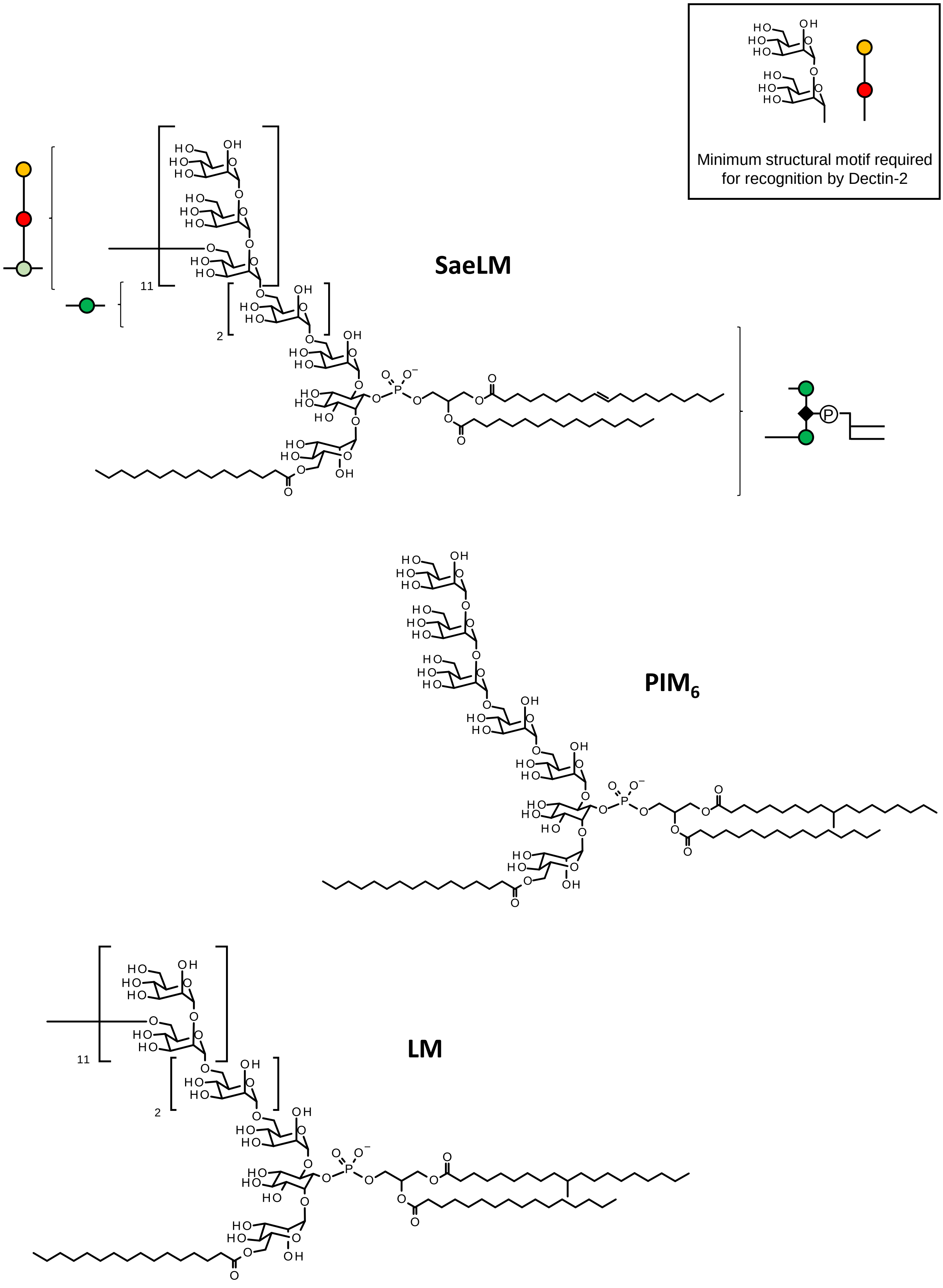
Minimum structural motif required for recognition by Dectin-2
SaeLM
PIM6
LM

**Fig. S1A**

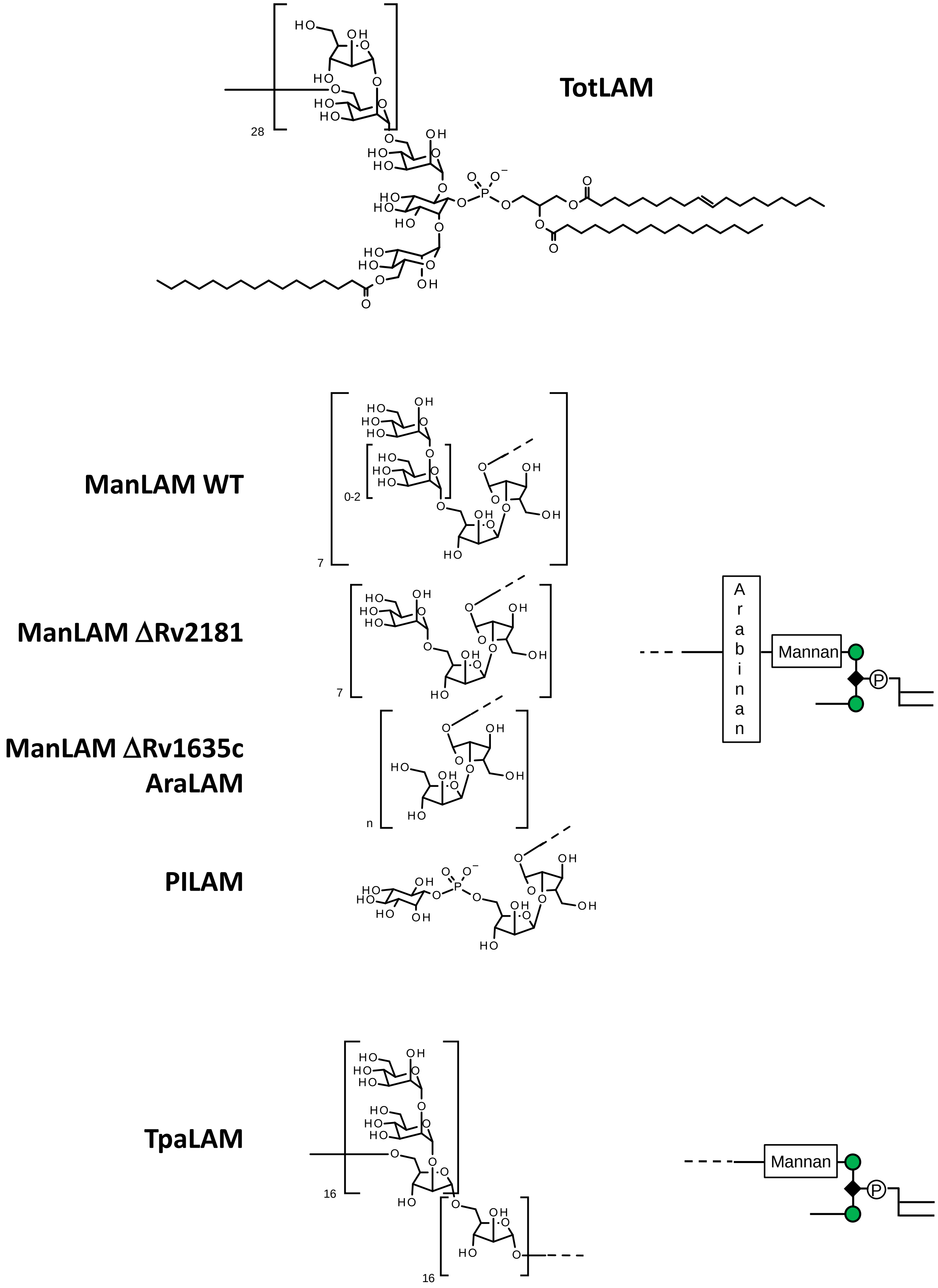


**Fig. S1A (continues)**

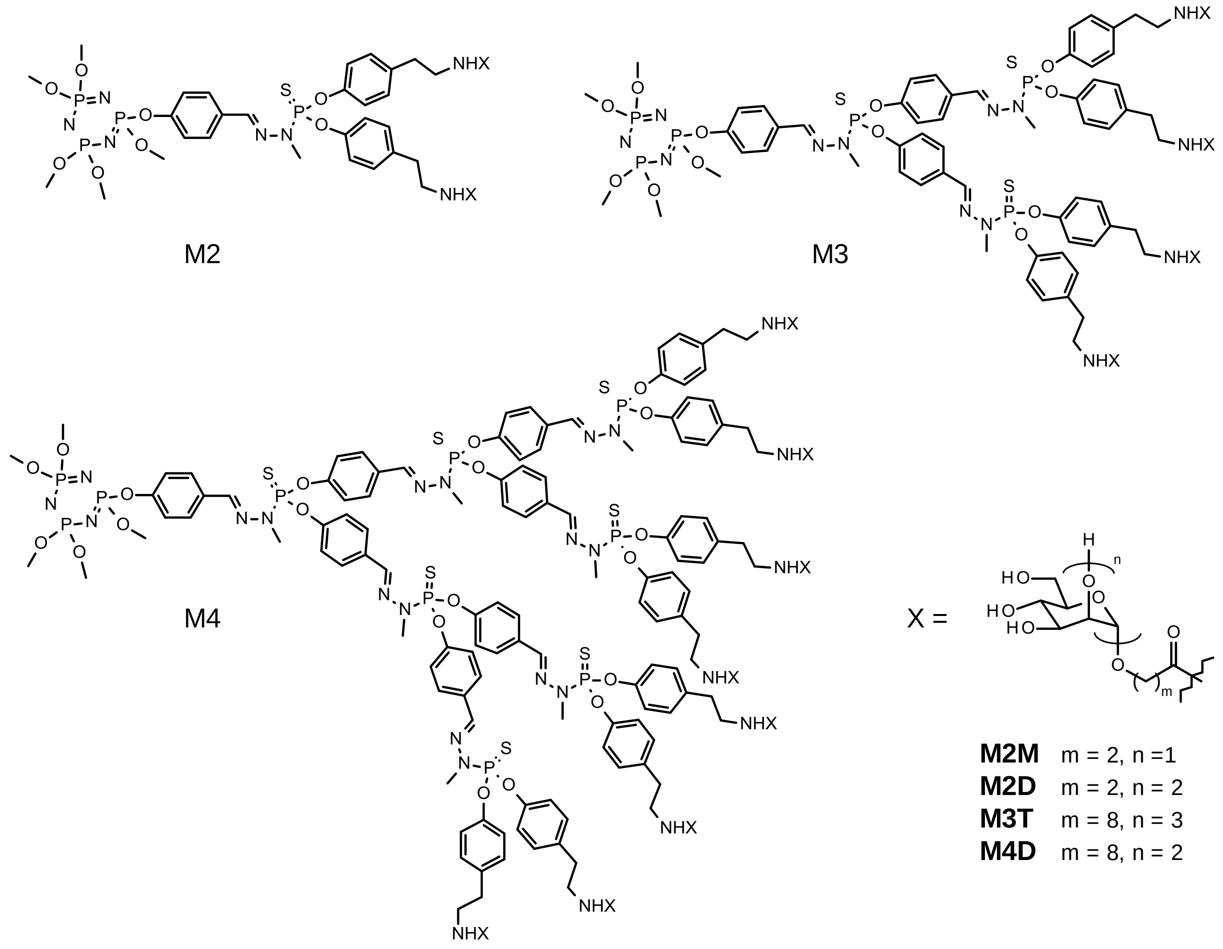


**Fig. S1B**

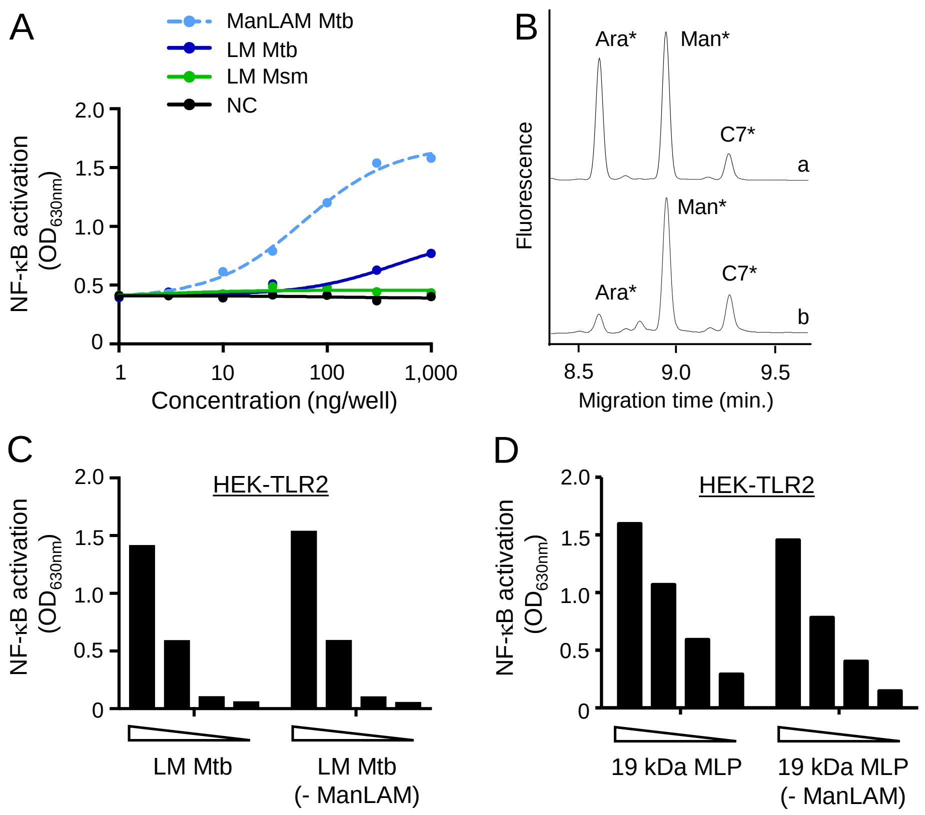

A
ManLAM Mtb
LM Mtb
LM Msm
NC
NF-κB activation (OD630nm)
2.0
1.5
1.0
0.5
0
1
10
100
1,000
Concentration (ng/well)
B
Ara*
Man*
C7*
a
Man*
Ara*
C7*
b
Fluorescence
8.5
9.0
9.5
Migration time (min.)
C
HEK-TLR2
LM Mtb
LM Mtb (- ManLAM)
D
HEK-TLR2
19 kDa MLP
19 kDa MLP (- ManLAM)


Fig S2

A

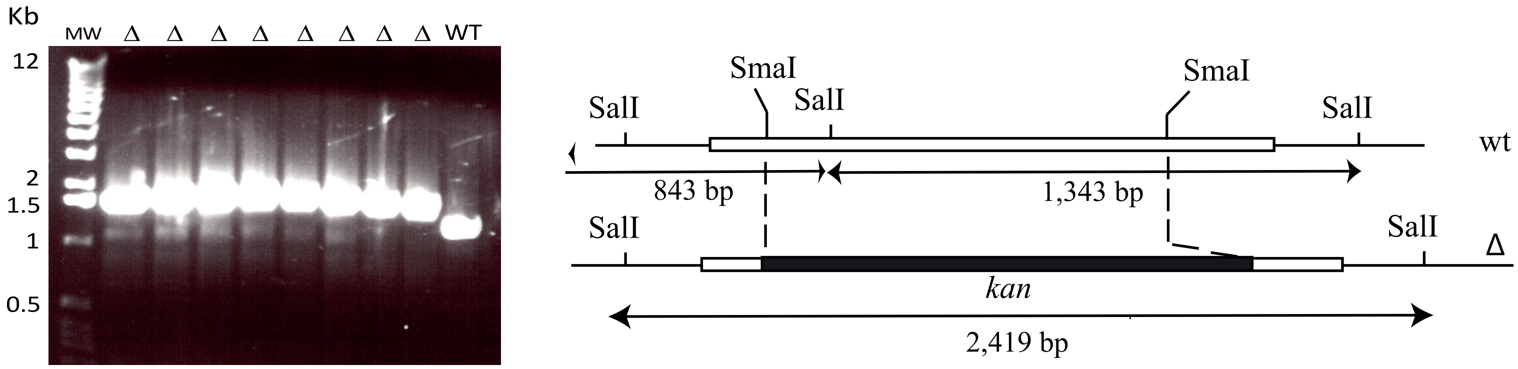

Kb
MW
Δ
Δ
Δ
Δ
Δ
Δ
Δ
Δ
WT
12
2
1.5
1
0.5
SmaI
SmaI
SalI
SalI
SalI
wt
843 bp
1,343 bp
SalI
SalI
Δ
kan
2,419 bp


B

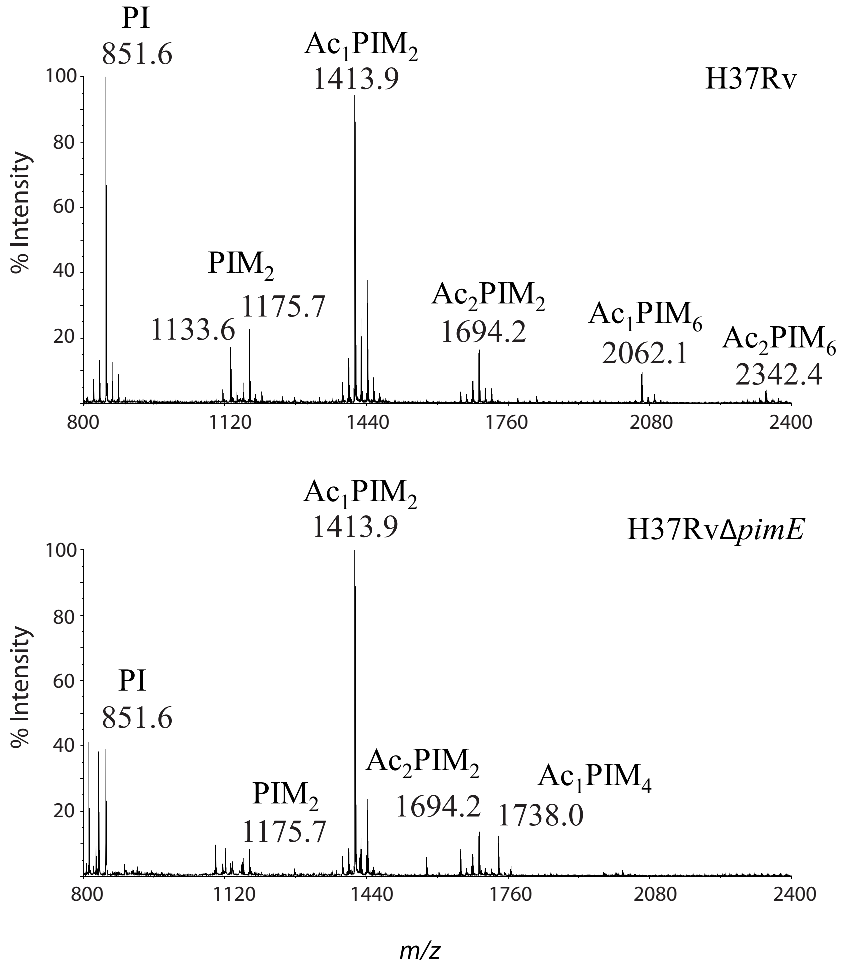

PI
851.6
$Ac_1PIM_2$
1413.9
H37Rv
100
80
60
40
20
% Intensity
$PIM_2$
1133.6
1175.7
$Ac_2PIM_2$
1694.2
$Ac_1PIM_6$
2062.1
$Ac_2PIM_6$
2342.4
800
1120
1440
1760
2080
2400
$Ac_1PIM_2$
1413.9
H37RvΔ*pimE*
100
80
60
40
20
% Intensity
PI
851.6
$Ac_2PIM_2$
1694.2
$Ac_1PIM_4$
1738.0
$PIM_2$
1175.7
800
1120
1440
1760
2080
2400
*m/z*


**Fig. S3**
